# Detection of a novel single nucleotide polymorphism in *IGF2* gene with a negative impact on egg production and body weight in Japanese quail (*Coturnix japonica*)

**DOI:** 10.1186/s43141-021-00271-7

**Published:** 2021-11-04

**Authors:** Dhafer A. Ali, Mohammed Baqur S. Al-Shuhaib, Golzar Farhadi, Fadhil R. Al-Kafajy, Tahreer M. Al-Thuwaini, Ali Esmailizadeh

**Affiliations:** 1 Department of Animal Production, College of Agriculture, Al-Qasim Green University, Al-Qasim, Babil 51001 Iraq; 2 Department of Animal Science, Faculty of Agriculture, Shahid Bahonar University of Kerman, Kerman, PB 76169-133 Iran

**Keywords:** Eggs production, Gene variation, *IGF2*, Japanese quails

## Abstract

**Background:**

Insulin-like growth factor 2 (*IGF2*) is one of three hormones that share high structural similarity to insulin. It is involved in several insulin-like growth-regulating and mitogenic activities. This study was conducted to genotype the coding regions of the *IGF2* gene in Japanese quail (*Coturnix japonica*) using PCR-SSCP-sequencing, and to assess the possible association of the polymorphism of these regions with the main egg production traits. A total of 240 female birds with an equal number of three Japanese quail populations (Brown or BR, Black or BL, and White or WT) were included in this study.

**Results:**

All the genotyped regions exerted no heterogeneity in their sequences with one exception detected in the exon 2. In this locus, a novel single nucleotide polymorphism (SNP) was detected in which “A” was substituted with “G” at 81 position with a silent effect (p.F79=SNP) on IGF2 protein. Association analyses indicated a significant (*P* < 0.05) relation of this SNP with egg number (EN) and bird weight (BW) in the analyzed populations, in which the birds with AG genotype had lower EN and BW than those with AA genotype. The p.F79=SNP was largely detected in the WT line than the other two lines.

**Conclusion:**

The detected p.F79=SNP has a highly negative effect on EN and BW in Japanese quail. Thus, the implementation of the variations of the *IGF2* gene can be a useful marker in the marker-assisted selection of Japanese quail. This is the first report to describe *IGF2* gene variations in Japanese quail, which strongly suggests raising the birds from the BR line with AA genotype when breeders desire to raise Japanese quail for large-scale egg production.

## Background

Japanese quails (*Coturnix japonica*) are the smallest farmed avian species with increasing profitable avenue as an alternative source for meat and eggs. These small-to-medium-sized birds belong to the same family of chicken and pheasant (Phasianidae) and have considerable similarities in physical characteristics and behavior [1]. However, these hardy birds characterize with more advantages than chickens, such as their substantial capacity to benefit from food, its resistance to many poultry diseases that invade chickens, low feed intake, and high reproductive proportions [2]. For these reasons, these birds are increasingly raised for producing both eggs and meats and as a laboratory animal model for multiple areas of scientific inquiries [3]. It is well-known that Japanese quails have achieved considerable importance in the specialty markets among agricultural species due to the unique flavor and high nutritional value of their meat and eggs [4, 5]. However, several strategies of quails breeding have still been out of focus since the breeding of these is being challenged with decreased and unbalanced productivity. Therefore, the issues of selecting the most favorable quail populations for egg production are mandatory. So that, several candidate genes have been selected and considered as a basis for market-associated selection and their association with the main productive traits has been analyzed [6, 7]. In addition, a number of genomic regions linked to the production traits in Japanese quail have been identified using linkage analyses [8–11]. One of these highly interesting candidates is the *IGF2* gene (gene ID; 107314538). Due to multiple growth regulating activities attributed to this crucial insulin-similar gene, it can be considered as one of the major candidates for breeding improvements in birds. It consists of four exons and three introns within chromosome number 5 in quails (NCBI Reference Sequence: NC_029520.1). The expressed product of the *IGF2* gene is the insulin growth factor 2 (IGF2), which is composed of 224 amino acids that share remarkable structural similarities with insulin (NCBI Reference Sequence: XP_015719328.1). IGF2 has a wide range of activities in controlling growth and development, such as cell growth promotion, survival control, migration, and differentiation via its corresponding IGF receptor [12]. It has been well established that this gene is involved in the stimulation of mitotic responses, and its knockout in mice leads to a significant reduction in body weight [13]. In birds, the versatile roles of IGF2 in controlling growth rate, lipid metabolism, and body composition have been documented [14]. The association of *IGF2* gene polymorphism with growth and production traits has extensively been described in chickens [15]. Despite the beneficial information obtained from these association studies, this possible association has not been described in Japanese quail. Therefore, the present study aimed to investigate the association between *IGF2* gene polymorphism with egg production traits in three domesticated lines of Japanese quail. According to our knowledge, this study is the first one to analyze the genetic variation of the *IGF2* gene in Japanese quail.

## Methods

### Japanese quail lines

Initially, 1200 eggs of Japanese quail (*Coturnix japonica*) were included in the study. Equal total number of these eggs were collected from three lines of quails; Brown (BR) line (*n* = 400), Black (BL) (*n* = 400), and White (WT) line (*n* = 400). Eggs were incubated and hatched using the hatching guidelines recommended by Romao et al. [16]. The living conditions, housing, feeding, temperature, sex determination were performed according to Al-Kafajy et al. [17]. Once quails get sexual maturity, males were excluded from the study, and only females were retained and individually maintained in laying cages. Subsequently, the egg number (EN), egg weight (EW), and birds weight (BW) were recorded on a total of 240 female birds from three lines of Japanese quail, BL (*n* 80), BR (*n* 80), and WT (*n* 80). All EN, EW, and BW traits were recorded weekly, starting from the proposed sexual maturity week (week 6) until week 12.

### Genomic DNA isolation

One drop of peripheral blood was collected from the wing vein of each female quail. Genomic DNA was extracted from all 240 birds using a manual salting out procedure [18]. The quantity and quality were described in birds [19]. The genomic DNA integrity was visualized by direct electrophoresis on ethidium bromide pre-stained 0.08% agarose evaluated using a Nanodrop spectrophotometric method (Biodrop, μLITE, UK).

### Polymerase chain reaction

Four PCR fragments were designed using NCBI Primer-BLAST software [20]. The main designing approach for these four primers pairs was made to cover all four coding regions of the *IGF2* gene in such a way each PCR amplicon would represent one specified exon. The lyophilized oligonucleotides were purchased from Bioneer (Daejeon, South Korea). The PCR amplification reactions were performed using Bioneer PCR premix. The optimum amplification conditions for the four designed amplicons were empirically determined using a gradient PCR as described in Table 1. Standard PCR experiments were performed on PCR thermocycler (Nexus, Eppendorf, Hamburg, Germany). The amplification protocol was initiated by one cycle of denaturation at 94°C for 4 min, followed by 30 cycles of denaturation at 94°C for 30 s, annealing for 45 s, and elongation at 74°C for 45 s, and was concluded with a final extension at 72°C for 5 min. PCR amplicons were verified by electrophoresis on 1.5% agarose gel.

**Table 1:** The oligonucleotide primer sets designed for the amplification of the *IGF2* in three populations of Japanese quails. The present annotations of this study variants were based on GenBank accession number NC_029520.1

Set	Primer code	Primer sequence (5′ → 3′)	Locus	Length	Annealing temp.
1	IGF2,exo1-F	GGTCCCTCTAGTGACACGC	Exon 1	365 bp	NA
	IGF2,exo1-R	CAAAGCGAGGAGAGAGAGCC			
2	IGF2,exo2-F	TTGGCATAGCATGAGGTGGG	Exon 2	232 bp	61.0 °C
	IGF2,exo2-R	ATGGCTTCTTTCCCCAGGTG			
3	IGF2,exo3-F	CTACCTTGTTGAGGGCTGGG	Exon 3	236 bp	60.7 °C
	IGF2,exo3-R	TGGGTAAAGGGTGACGAAGC			
4	IGF2,exo4-F	CCGGCTGGTCACAGTTCATT	Exon 4	277 bp	59.8 °C
	IGF2,exo4-R	GTTGTTCTCCCTTCCCCAGG			

### Single-strand conformation polymorphism (SSCP)

SSCP experiments were performed using a rapid high voltage approach [21], with several modifications. Briefly, each PCR amplicon was treated with an equal volume of SSCP denaturing-loading buffer (95% formamide, 0.05% xylene cyanol, 0.05% bromophenol blue, and 20mM EDTA, pH 8). After denaturation for 7 min, 2 μl of PCR amplicons were immediately placed on ice and frozen for about 10 min. Subsequently, 2 μl of samples were loaded on neutral polyacrylamide gel (0.1 mm thickness, 10 cm length, and 20 cm width). Electrophoresis conditions were optimized according to details described in Table 2. Gels were fixed and stained according to the protocol of Byun *et al*. [22].

**Table 2:** SSCP electrophoresis conditions of the amplicons of the *IGF2* gene in three lines of Japanese quails. All PCR amplicons were electrophoresed on 0.1 mm thickness of 10 × 20 (L × W) cm diameters polyacrylamide gels

Set	Amplicons	Gel concentration	Running temperature	Running time	Running voltage	Running amperage
1	IGF2,exo2	8%	16°C	4.0 hr	210V	100 mA
2	IGF2,exo3	10%	18°C	4.5 hr	200V	100 mA
3	IGF2,exo4	8%	20°C	4.5 hr	200V	105 mA

### DNA sequencing

Each detected genotype on the SSCP gel was subsequently exposed to sequencing reaction from both termini according to instructions of Macrogen laboratories (Macrogen, Geumchen, Seoul, Korea). The referring database of the *IGF2* nucleic acid sequences was retrieved (https://www.ncbi.nlm.nih.gov). The sequenced SSCP genotypes were visualized and annotated by BioEdit ver, 7.1. (DNASTAR, Madison). The amino acid reading frames of the SSCP variants were determined using the Expasy translate server (http://web.expasy.org/translate/). Each detected variant was visualized using SnapGene Viewer ver. 4.0.4 (http://www.snapgene.com). Only clear electropherograms were considered in the observed variations in comparison with their retrieved corresponding references of the IGF2 sequences (GenBank accession number NC_029520.1). The appropriate reading frame of each observed variant was aligned with its corresponding reference sequences within the IGF2 protein using the UniProtKB server (http://www.uniprot.org/align/).

### Genetic diversity analyses

The assessment of the genetic polymorphism of the *IGF2* gene variants was performed by calculating allele and genotype frequencies, observed heterozygosity (*Ho*), expected heterozygosity (*He*), and the effective number of alleles (*Ne*) were performed using PopGen32 software, v. 1.31 [23]. Chai-squared test (*χ*^2^) was also calculated to verify the possible deviation from Hardy–Weinberg Equilibrium (HWE) expectations for the distribution of genotypes. The polymorphism information content (PIC) was computed using the following formula [24].

$$\mathrm{PIC}\kern0.5em =\kern0.5em 1\hbox{-} {\sum}_{i=1}^m{p}_i^2-{\sum}_{i=1}^{m-1}{\sum}_{j=i+1}^m\kern0.5em 2{p}_i^2{p}_j^2$$

where *pi* and *pj* are the frequencies of the *i*th and *j*th allele, respectively, and m is the number of alleles.

### Association analyses

Statistical analyses to determine the significance of the lines and IGF2 genotypic effects were *analyzed by ANOVA-repeated measures (GLM procedure of SPSS, v 23), in which* the following model was used;

$${Y}_{ij k}=\kern0.5em \mu +{L}_j+{G}_k+{(LG)}_{jk}+{p}_j+{\gamma}_{ij}++{\eta}_{ik}+{e}_{ij k}$$

where *μ* is the overall mean, *L*_*j*_ is the main effect of lines to (∑ *L*_*j*_=0), *G*_*k*_ is the main effect of genotype to (∑ *G*_*k*_=0), (*LG*) _*jk*_ is the interaction effect, *p*_*j*_ is the main effect of subjects *N* (0, σ^2^_*i*_), *γ*_*ij*_ is the interaction effect of subjects and lines *∼ N* (0; σ^2^_ij_), *ƞ*_*ik*_ is the interaction effect of subjects and genotype *∼ N* (0; σ^2^_ik_) and *e*_*ijk*_ is random error assumed *e*_*ijk*_ *∼ N* (0; σ^2^). Means of the significant main factors were compared using the Tukey test. *P*-values of less than 0.05 were considered statistically significant for all comparisons.

## Results

### Genotyping analyses

**Fig. 1:**
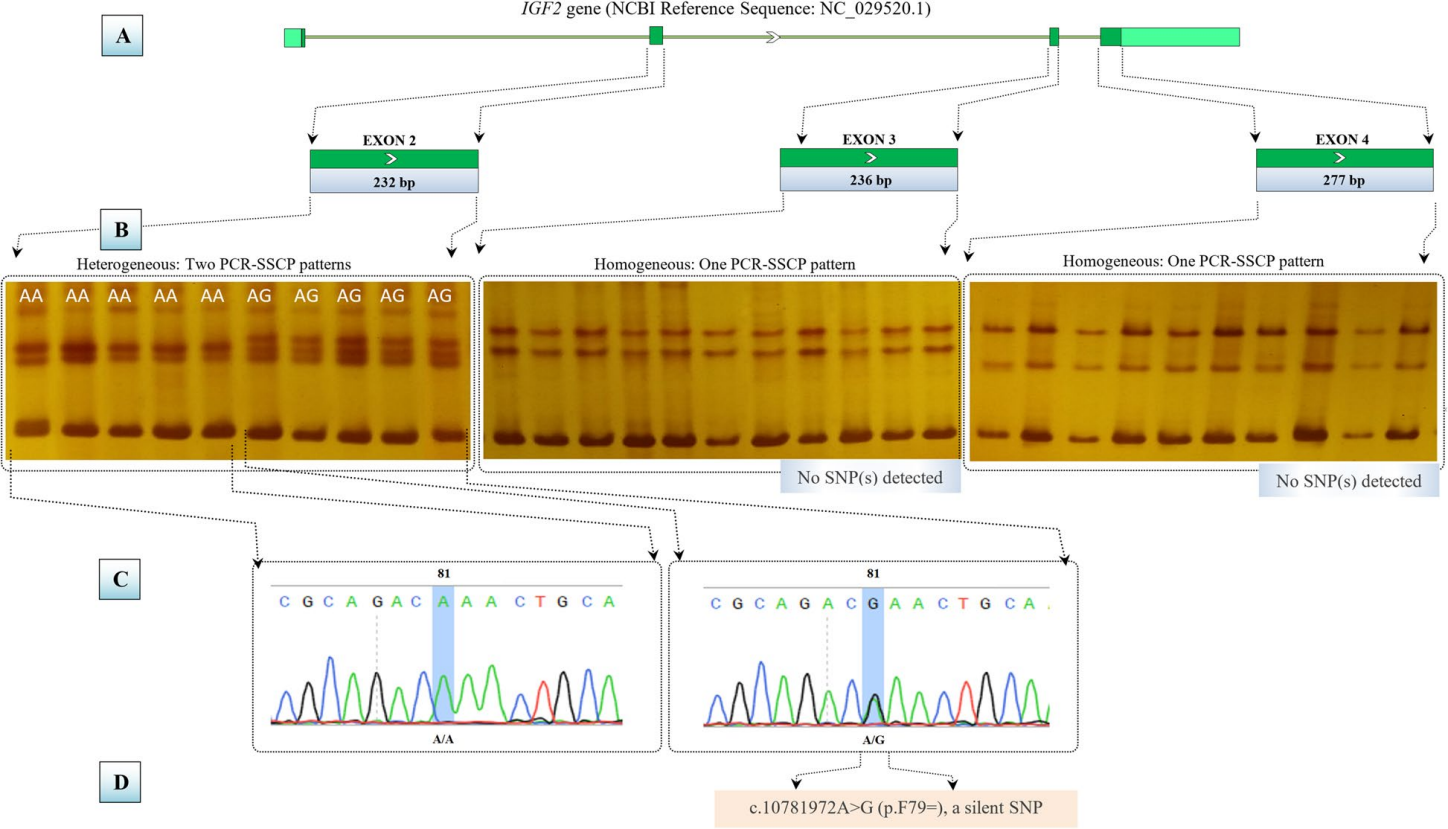
A schematic diagram for the *IGF2* gene-based PCR-SSCP-sequencing strategy in three lines of Japanese quails. **A** PCR design of three PCR-specific primers pairs for the amplification of 232 bp, 236 bp, and 277 bp in exon 2, exon 3, and exon 3, respectively. **B** Post-PCR genotyping using SSCP technique, in which only exon 2 showed two different genotypes. **C** Sequencing reactions interpretation of the detected genotypes, in which only one SNP was detected in the exon 2. **D** Characterization of the observed SNP, in which a silent effect p.F79= was observed in the detected SNP. The positions of primers and the nomenclature of the observed SNPs are listed according to NCBI Reference Sequence: NC_029520.1 following the nomenclature described in varnomen.hgvs.org/

All the coding regions of the *IGF2* gene, as well as their flanking regions, were screened by designing four specific PCR primers (Fig. 1A). Unfortunately, the PCR experiments of the first PCR amplicons designed to specifically amplify the exon 1 were tuned to be problematic since no specific results were detected. The other three amplicons of exon 2, exon 3, and exon 4 amplicons showed specific PCR products, of 232 bp, 236 bp, and 277 bp, respectively. Concerning both exon 3 and exon 4 amplicons, no heterogeneity was observed after being analyzed by PCR-SSCP gels. Considering exon 2 amplicons, two PCR-SSCP patterns were detected, one pattern with two bands and the other one with three bands (Fig. 1B). Sequencing reactions confirmed this heterogeneity by detecting a homozygous A/A electropherograms at the 81th position in the two bands pattern and a heterogeneous A/G electropherograms at the same position in the three bands pattern (Fig. 1C). The novelty of the observed A81G SNP was confirmed by Ensembl browser 9. Both detected SSCP genotypes were assigned AA and AG for the homogenous and heterogeneous status of each genotype. The observed AA and AG genotypes were respectively deposited in the NCBI-bankit database under the accession numbers (MT193259 and MT193260). It was observed that this SNP was positioned in the Phe79 within the IGF2 protein. The silent effect (p.F79=) was determined by expasy translate and UniprotKB servers, respectively (Fig. 1D).

### Genetic diversity and association analyses

Concerning the BR line, the most abundant genotype was AA, which was predominantly observed in this studied population with a total frequency of 0.76 (*n* = 61). The highly dominant AA genotype was followed by the AG genotype, which was detected with a total frequency of 0.24 (*n* = 19). The high percentage of AA genotype was reduced in the BL line, which was detected in lower frequency (0.65, *n* = 52), with an increased ratio of AG genotype (0.35, *n* = 28). In contrast to BR and BL lines, the lowest frequency of AA genotype (0.34, *n* = 27) was observed in the WT line, with the advent predominance of the AG genotype (0.66, *n* = 53). This sort of genotype distribution ranged from low in the BR line (PIC = 0.188), intermediate in the BL line (PIC = 0.251), to high in the WT line (PIC = 0.344) (Table 3). However, the values of *Ho*, *He*, *Ne*, and PIC in WT line at p.F79= SNP locus were higher than that of BL and BR breed, respectively, which implied that the polymorphism and genetic variation of WT breed were higher than that found in BL and BR populations, respectively. However, the Chai square tests showed that the polymorphism of the *IGF2* gene in all three studied birds populations showed significant deviation from the Hardy Weinberg equilibrium (*P* < 0.05).

**Table 3:** Genotype and allele frequencies and genetic diversity variables for p.F79= SNP locus of the *IGF2* gene in three lines of Japanese quails

	Genotype frequencies (*n*)		Allele frequencies						
Line	AA	AG	A	G	***Ho***	***He***	***Ne***	***χ***2	***PIC***
BR	0.76 (*n* = 61)	0.24 (*n* = 19)	0.8812	0.1187	0.2375	0.2106	0.2093	1.368693	0.188
BL	0.65 (*n* = 52)	0.35 (*n* = 28)	0.8250	0.1750	0.3500	0.2906	0.2888	3.453851	0.251
WT	0.34 (*n* = 27)	0.66 (*n* = 53)	0.6687	0.3312	0.6625	0.4458	0.4430	19.196262	0.344

*n* number of samples, *χ*^2^ chi-square, *Ho* observed heterozygosity, *He* expected heterozygosity, *Ne* effective allele number, *PIC* polymorphism information content. All Chi-square tests have one degree of freedom and are within the significance level *P* < 0.05

Association analyses results showed a significant (*P* < 0.05) contribution of lines of birds (BR, BL, and WT) and the two detected genotypes (AA and AG genotype) of the *IGF2* gene. This line/genotype interaction was continuously observed in two measured production traits at all time intervals. Line of birds and genotype of *IGF2* were both significantly (*P* < 0.05) associated with EN and BW in all recorded egg production weeks (Table 4). Differences between BR, BL, and WT lines were statistically significant (*P* < 0.05) for EN and BW traits, since birds with AA genotype showed higher EN and BW traits than birds with AG genotype. This observation implied that the silent p.F79= SNP of the AG genotype exerted a negative significant (*P* < 0.05) association with EN and BW traits. The remarkable differences in the frequency of AA genotype (0.76) than AG genotype had given the BR line advent superiority in terms of egg production traits than BL and WT lines, respectively. However, no significant (*P* < 0.05) association between line/genotype factors was observed in correlation with EW in the entire measurement period. Nevertheless, a clear tendency of birds with BR lines to exhibit more values than the BL and WT lines respectively was observed in almost all measured EW. The same tendency of birds with AA genotype to exhibit higher values in EW than birds with AG genotypes was also observed. However, these differences were not reached the significant thresholds in all recorded weeks (Table 5).

**Table 4:** Least square means (±SE) of interaction effects of AA and AG genotypes on egg production traits in three lines of Japanese quails

Time (week)	Trait	BR		BL		WT	
		AA	AG	AA	AG	AA	AG
Week 6	**EN**	49.617± 3.541^A a^	40.563± 2.529 ^B c^	45.096± 1.283 ^A a b^	39.871± 2.548 ^B c^	42.134± 3.567 ^A b^	39.096± 2.522 ^B c^
	**EW**	9.883± 0.545	9.794± 0.504	9.605± 0.559	9.489± 0.502	9.526± 0.499	9.403± 0.550
	**BW**	218.091± 10.551 ^A a^	211.094±11.504 ^B b^	211.265±11.263 ^A b^	206.389±12.502 ^B a b^	204.126±13.499 ^A a b^	200.103±11.550 ^B c^
Week 7	**EN**	59.654±2.659 ^A a^	55.694±3.504 ^B b^	55.742±2.535 ^A b^	52.682±4.541 ^B a b^	51.400±5.518 ^A a b^	49.417 ±3.541 ^B c^
	**EW**	10.887±0.558	10.652±0.536	10.607±0.556	10.489±0.502	10.430±0.520	10.401±0.540
	**BW**	224.225±13.809 ^A a^	214.294±10.504 ^B b^	220.409±9.985 ^A a^	210.489±8.502 ^B c^	215.503±12.513 ^A b^	210.515±10.543 ^B c^
Week 8	**EN**	75.151±1.301 ^A a^	70.294±2.504 ^B b^	68.892±2.591 ^A a b^	62.878±3.535 ^B c^	65.557±2.185 ^A a b^	61.198±1.618 ^B c^
	**EW**	10.995±0.552	10.763±0.533	10.996±0.558	10.703±0.511	10.726±0.523	10.603±0.539
	**BW**	229.621±10.523 ^A a^	219.794±11.504 ^B b^	219.371±10.226 ^A b^	210.489±8.489 ^B c^	212.526±7.499 ^A c^	208.503±9.503 ^B c^
Week 9	**EN**	69.261±1.232 ^A a^	65.394±3.504 ^B b^	64.590±2.586 ^A b^	61.582±2.546 ^B c^	62.603±3.503 ^A c^	60.615±5.548 ^B c^
	**EW**	11.888±0.554	11.794±0.509	11.701±1.566	11.689±1.502	11.834±0.492	11.600±0.552
	**BW**	226.259±10.638 ^A a^	216.294±9.504 ^B b^	224.257±12.693 ^A a^	214.589±10.502 ^B b^	216.526±11.499 ^A b^	206.503±10.550 ^B c^
Week 10	**EN**	72.869±1.805 ^A a^	67.136±2.553 ^B b^	66.515±3.833 ^A b^	63.385±2.542 ^B a b^	62.007±3.465 ^A a b^	59.013±2.564 ^B c^
	**EW**	11.387±0.558	11.194±0.504	11.296±0.579	11.289±0.502	11.326±0.523	11.103±0.539
	**BW**	230.656±11.335 ^A a^	228.494±10.504 ^B b^	226.588±13.184 ^A b^	220.989±12.502 ^B a b^	219.326±10.499 ^A a b^	209.303±9.550 ^B c^
Week 11	**EN**	79.585±1.065 ^A a^	75.694±2.504 ^B b^	70.400±2.610 ^A a b^	69.353±2.536 ^B a b^	67.342±4.539 ^A a b^	62.262±5.529 ^B c^
	**EW**	11.987±0.558	11.794±0.504	11.605±0.559	11.589±0.502	11.626±0.499	11.503±0.550
	**BW**	234.567±7.754 ^A a^	231.494±9.504 ^B a^	231.117±12.979 ^A a^	218.489±10.502 ^B a b^	225.326±11.499 ^A b^	211.303±9.550 ^B c^
Week 12	**EN**	79.182±3.087 ^A a^	74.294±2.504 ^B b^	71.625±3.083 ^A b^	69.775±3.537 ^B a b^	68.973±2.519 ^A a b^	62.030±4.540 ^B c^
	**EW**	11.988±0.555	11.694±0.504	11.705±0.559	11.589±0.502	11.526±0.499	11.503±0.550
	**BW**	233.030±6.626 ^A a^	230.036±5.553 ^B a^	230.198±10.480 ^A a^	218.471±8.482 ^B a b^	223.026±10.499 ^A b^	213.003±7.550 ^B c^

Values followed by different alphabets differ significantly

^A, B^different capital letters indicate a significant difference in the genotypes within each classification (*P* ≤ 0.05).

^a, b^Different lowercase letters indicate a significant difference in lines within each classification (*P* ≤ 0.05)

EN, EW, and BW refer to egg number, egg weight, and bird weight, respectively. Data expressed as means±SD

**Table 5:** Least square means (±SD) of the main effects of AA and AG genotypes and lines on egg production traits in three lines of Japanese quails

Time (week)	Trait	Line			Genotype	
		BR	BL	WT	AA	AG
Week 6	**EN**	49.108±3.534^a^	44.017±3.017 ^a b^	38.604±4.535 ^b^	47.512±4.940 ^a^	41.120±3.998 ^b^
	**EW**	9.811±1.530	9.700±1.537	9.685±2.532	9.760±0.529	9.510±0.550
	**BW**	217.092±10.538 ^a^	211.308±11.058 ^a b^	204.111±10.530 ^b^	209.862±12.885 ^a^	200.472±13.634 ^b^
Week 7	**EN**	59.664±5.623 ^a^	54.721±4.534 ^a b^	51.411±4.530 ^b^	58.897±4.070 ^a^	50.984±3.984 ^b^
	**EW**	10.811±2.530	10.601±1.535	10.479±1.550	10.242±0.528	10.021±0.549
	**BW**	224.242±10.746 ^a^	222.437±10.845 ^a^	215.511±10.530 ^b^	221.188±13.328 ^a^	211.576±13.549 ^b^
Week 8	**EN**	70.185±3.163 ^a^	65.887±3.569 ^a b^	61.316±2.818 ^b^	69.487±4.377 ^a^	61.117±3.713 ^b^
	**EW**	10.787±0.545	10.698±1.539	10.611±1.530	10.727±0.549	10.662±0.529
	**BW**	229.661±11.354 ^a^	219.412±11.029 ^a b^	212.511±10.530 ^b^	222.639±16.817 ^a^	210.745±16.611 ^b^
Week 9	**EN**	69.292±3.104 ^a^	64.537±2.569 ^a b^	60.611±3.530 ^b^	67.804±4.106 ^a^	61.554±3.398 ^b^
	**EW**	11.990±0.541	11.900±0.541	11.811±0.530	11.920±0.548	11.871±0.532
	**BW**	226.267±10.607 ^a^	219.373±12.190 ^a b^	206.511±10.530 ^b^	221.851±17.592 ^a^	211.328±19.449 ^b^
Week 10	**EN**	72.932±3.602 ^a^	66.470±2.743 ^a b^	60.011±3.530 ^b^	69.006±5.747 ^a^	61.791±4.353 ^b^
	**EW**	11.098±0.543	11.093±0.550	11.011±0.530	11.805±0.584	11.003±0.572
	**BW**	230.728±12.582 ^a^	220.618±11.192 ^a b^	209.311±10.530 ^b^	226.241±17.932 ^a^	217.502±19.456 ^b^
Week 11	**EN**	79.611±3.961 ^a^	74.383±3.582 ^a b^	71.288±2.530 ^b^	76.571±3.840 ^a^	69.920±3.593 ^b^
	**EW**	11.988±0.543	11.700±0.537	11.611±0.530	11.601±0.544	11.498±0.523
	**BW**	231.247±12.418 ^a^	226.550±10.701 ^a b^	211.311±10.530 ^b^	225.427±17.309 ^a^	219.842±19.282 ^b^
Week 12	**EN**	78.208±3.980 ^a^	71.677±3.928 ^a b^	66.011±3.530 ^b^	76.479±4.763 ^a^	69.699±4.775 ^b^
	**EW**	11.990±0.541	11.600±0.537	11.411±0.530	11.557±0.547	11.479±0.525
	**BW**	231.293±12.016 ^a^	226.032±10.607 ^a b^	213.011±10.530 ^b^	229.792±16.025 ^a^	218.091±17.670 ^b^

Values followed by different alphabets differ significantly

^a, b^Different lowercase letters indicate a significant difference within each classification (*P* ≤ 0.05)

EN, EW, and BW refer to egg number, egg weight, and bird weight, respectively

## Discussion

Several reasons led us to conduct the present study on the *IGF2* gene in Japanese quails. It was reported that IGF2 polypeptide is a member within the somatotropic axis with advent ability to regulate growth and development, and thus egg production trait in several breeds of chickens [15]. Besides, polymorphism in the *IGF2* gene was tightly linked with egg production traits in Muscovy ducks [25]. These observations may refer to a considerable correlation between IGF2 function and egg production. Likewise, several candidate genes are increasingly being reported to be associated with production traits in quails [26–28]. For this reason, the genetic variations within this critical gene may bring several alterations for productive performance in quail populations.

After screening Japanese quails to investigate the *IGF2* polymorphism, two genotypes were detected, AA genotype and AG genotype. One silent, p.F79=SNP was detected in birds with AG genotype. Since birds with AA genotype were characterized with better production traits than those with AG genotype, obvious negative impacts of the p.F71= SNP on these traits were observed. This observation entails a remarkable association between this SNP and the low values of EN, BW, and EW to a little extent. However, this finding may signify a considerable importance for this silent SNP as a causative factor for metabolic alterations [29]. Though no missense SNP was detected in all the investigated quail’s population, the present study detected a crucial silent SNP, which was found to be associated with low productivity in quails. This finding may add a further layer of confirmation on the recent understanding of the critical functionality of the silent SNP in various metabolic activities [30]. Though there is no accurate mechanism to explain how silent SNP may have such a functional impact [31], it could change substrate specificity which may lead to alternative protein kinetics [32].

Apart from genotyping results, three lines of Japanese quails were investigated in this study, BR, WT, and WT. BR line showed higher egg production values than BL and WT lines respectively. In all measured production weeks, birds of BR line had shown higher values of EN and BW than both birds of BL and WT lines respectively. In agreement with our findings, Bagh *et al* [33] have also revealed the superiority of BR birds over other birds in terms of egg production traits. However, each selected line has been found to exhibit a unique association with egg production [17]. In addition to the observed differences in plumage color among BR, BL, and WT lines [34], it has been reported that each line is characterized by a different productive performance [35]. Due to these considerable differences among these lines, it is rational to explain clear intervention of all three lines as non-genetic factors alongside the observed genetic factors with the measured production traits in Japanese quails [36]. However, it is well established that production in birds is multifactorial and it could not be regulated by implicating only one factor, whether being based on genetic or non-genetic origin. Nevertheless, our conducted genetic diversity analyses indicated three distinct frequencies of the detected *IGF2*-based genotyping in the three investigated populations. BR line exhibited the lowest AG genotype and highest AA genotype frequencies among the other two populations. In contrast to BR line, WT line exhibited the opposite distribution of both AA and AG genotypes, while BL line showed moderate distributions of them. Statistical analyses indicated that birds having AG genotype exerted the lower EN and BW than those with the AA genotype. This observation signifies that the line with a higher frequency of AG genotype had lower egg production traits. Taking these data into account, it can be concluded that BR line is the most recommended population for egg production since it showed a lower frequency of AG genotype than both BL and WT lines respectively. Accordingly, the results of this study indicated that both genetic (AA and AG genotypes) and non-genetic (BR, BL, and WT lines) factors were not found to exhibit an independent effect on the measured productive performance in the investigated lines of Japanese quails. Instead, a tight interaction between both factors was witnessed, in which BR line with AA genotype showed the best production values than BR line with AG genotype or other genetic or non-genetic formulas. Unfortunately, no association study between *IGF2* polymorphism and phenotypes traits was published in Japanese quails. Therefore, it can be stated that this study is the first report to describe such association, which pave the way for further understanding of *IGF2* polymorphism in other populations with special emphasis on the critical role of p.F71= in controlling EN and BW traits.

## Conclusion

In this study, the association of polymorphisms at the *IGF2* gene with egg production traits in three lines of Japanese quail is described. Advent interaction of both *IGF2* genotypes (AA and AG) and birds line (BR, BL, and WT) in association with EG and BW was observed. Considering the *IGF2* gene effect, it was found that birds with AG genotype, having the silent p.F71= SNP, had lower EN and BW than birds with AA genotype. Concerning the line effect, BR line showed the most favorable production values than both BL and WT lines. Accordingly, BR line with AA genotype was highly recommended for egg production purposes. Therefore, it can be stated that the polymorphism of the *IGF2* gene has the potential to be used in marker-assisted selection in Japanese quail. Because of the negative impact of p.F71= SNP on the measured traits, this study suggests confirming the absence of the p.F71= SNP before raising any Japanese quail population for large scale egg production.

## Data Availability

All data generated or analyzed during this study are included in this published article.

## Abbreviations

He: Expected heterozygosity
Ho: Observed heterozygosity
HWE: Hardy–Weinberg Equilibrium
IGF2: Insulin-like growth factor 2
PIC: Polymorphism information content
PCR: Polymerase chain reaction
SNP: Single nucleotide polymorphism
SSCP: Single-strand conformation polymorphism
